# Toxicity of Cultured Bullseye Puffer Fish *Sphoeroides annulatus*

**DOI:** 10.3390/md10020329

**Published:** 2012-02-03

**Authors:** Erick J. Nuñez-Vazquez, Armando Garcia-Ortega, Angel I. Campa-Cordova, Isabel Abdo de la Parra, Lilia Ibarra-Martinez, Alejandra Heredia-Tapia, Jose L. Ochoa

**Affiliations:** 1 Centro de Investigaciones Biologicas del Noroeste (CIBNOR), Apdo. Postal 128, La Paz, B.C.S. 23000, Mexico; Email: angcamp04@cibnor.mx (A.I.C.-C.); libarra04@cibnor.mx (L.I.-M.); 2 Investigacion para la Conservacion y el Desarrollo (INCODE), Andador 2, 245. Col. Banobras. La Paz, B.C.S. 23080, Mexico; Email: herediatap@yahoo.com.mx; 3 College of Agriculture, Forestry and Natural Resource Management, Pacific Aquaculture & Coastal Resources Center, University of Hawaii at Hilo, 200 W. Kawili St., Hilo, HI 96720, USA; Email: agarciao@hawaii.edu; 4 Centro de Investigacion en Alimentacion y Desarrollo (CIAD), Unidad Mazatlan. Apdo. Postal 711, C.P. 82010. Mazatlan, Sinaloa 82010, Mexico; Email: abdo@ciad.mx

**Keywords:** tetrodotoxin, toxicity, cultured puffer fish, *Sphoeroides annulatus*, food safety

## Abstract

The toxin content in various life cycle stages of tank-cultivated bullseye puffer (*Sphoeroides annulatus*) were analyzed by mouse bioassay and ESI-MS spectrometry analysis. The presence of toxin content was determined in extracts of sperm, eggs, embryo, larvae, post-larvae, juvenile, pre-adult, and adult fish, as well as in food items used during the cultivation of the species. Our findings show that only the muscle of juveniles, the viscera of pre-adults, and muscle, liver, and gonad of adult specimens were slightly toxic (<1 mouse unit). Thus, cultivated *S. annulatus*, as occurs with other cultivated puffer fish species, does not represent a food safety risk to consumers. This is the first report of toxin analysis covering the complete life stages of a puffer fish under controlled conditions.

## 1. Introduction

Bullseye puffer fish (*Sphoeroides annulatus* Jenyns, 1842), known in Mexico as “botete Diana”, is consumed in the northwestern and some central states of Mexico [1,2,3,4]. About 600 tons are exported annually, making Mexico the second largest exporter of puffer fish [5]. According to previous work [5], wild *S.* *annulatus* specimens are toxic in various tissues, including the liver (22 µg tetrodotoxin (TTX)/g), gonads (0.46 µg TTX/g), and intestine (0.42 µg TTX/g), but not in the muscles. Therefore, the fillet is edible and safe when prepared by a qualified cook. 

In Mexico, 37 human casualties have been linked to puffer fish consumption in the last 40 years [5,6]. Puffer fish toxicity is attributed to tetrodotoxin (TTX) and its analogs with varying degrees of toxicity [7,8]. The lethal dose of TTX for human is 0.5–2.0 mg [8]. This relates to a particular toxin profile, which is apparently determined by different environmental factors and food sources for each species of puffer fish [9,10,11]. Hence, the toxicity of puffer fish collected in different regions may vary significantly and its determination is a life-saving issue. Proper guidelines to regulate consumption of toxic puffer fish species have contributed to reduced casualties in several countries [12]. *Takifugu rubripes* (torafugu) and *T. niphobles* (kusafugu) have been successfully cultured in Japan, Korea, and China, where the contain low to no toxicity [10,11,13,14,15,16,17,18,19,20], demonstrating that toxicity of cultured puffer fish depends largely on their diet. This makes cultivation an attractive economic activity. In Mexico, the techniques for production of *S.* *annulatus* in fish hatcheries were developed in the past decade [21,22,23,24] and studies of the digestive tract ontogeny and capacity for digestion of food during larval development have been reported [25]. With the increasing interest in this species as a candidate for marine aquaculture in the tropical Eastern Pacific and potential toxicity issues, it is important to determine if this puffer fish is rendered safe and non-toxic under controlled conditions of cultivation.

## 2. Results and Discussion

**Figure 1:**
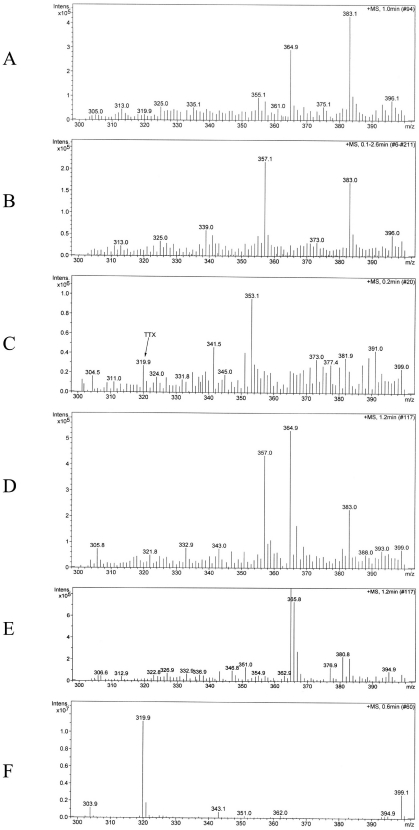
ESI mass spectra analysis of *Sphoeroides* *annulatus* at various developmental stages and of the feed used during cultivation: (**A**) juvenile (viscera); (**B**) pre-adult (muscle); (**C**) pre-adult (viscera); (**D**) *Artemia* nauplii; (**E**) Artificial feed; (**F**) TTX standard (319.9 *m/z*).

The results showed that the MBA found the presence of toxins in some extracts of cultivated *S. annulatus*, but in very low quantities (lower than 1 MU; less than 0.2 µg TTX/g of tissue). Only the viscera extracts of juvenile and pre-adult puffer fish, the muscles of juveniles, and the muscles, liver, and gonad of adult organisms induced mild signs of toxicity in mice (Table 1). Toxicity was indicated by rear limb paralysis and mild dyspnea. The most severe symptoms were caused by the viscera extract of pre-adult puffer fish and the liver of the adult, but all mice recovered within 1–2 h. Presence of TTX in samples of puffer fish and in *Artemia* nauplii, micro-particulate feed, and commercial feeds was also tested by ESI-MS analysis, indicating that whole extracts of sperm, eggs, embryo, larvae, post-larvae, and the feeds lack the peak at 319.9 *m/z* that correspond to TTX (Figure 1). In contrast, viscera tissue of pre-adults did show a peak at 319.9 *m/z*, indicating the presence of TTX (Figure 1).

The lack of toxicity in pre-adult muscle and the presence of TTX in pre-adult viscera confirmed our previous findings [5], suggesting that the toxin is redistributed among tissues during fish development.

Noguchi and Arakawa [11] collected more than 6000 specimens of *T. rubripes* from eight sites in Japan from 1981 through 2003. Specimens cultivated with nontoxic diets in net cages at sea or in aquaria were nontoxic, the liver toxicity never exceeded 2–10 MU/g, using an accepted TTX bioassay. Ji *et al.* [26] reported 565 nontoxic puffer fish cultivated in farms and net cages at sea. Specimens were tested during a year, based on a mouse bioassay for TTX.

**Table 1:** Toxicity of cultured puffer fish *Sphoeroides annulatus* and feed analyzed for tetrodotoxin (TTX) content.

Sample	Tissue/Characteristics	Mouse Bioassay Equiv. (µg/g)	Clinical Signs	Detection of TTX (ESI-MS)
Sperm	Complete	_	_	_
Eggs	Complete	_	_	_
Embryo	Complete	_	_	_
Larvae	Complete	_	_	_
Post-larvae	Complete	_	_	_
Juveniles	Muscle	_	+ ^a^	+
	Viscera *	_	+ ^a^	-
Juveniles	Muscle	_	+ ^a^	+
	Viscera *	_	+ ^a^	-
Pre-adults	Muscle	_	_	_
	Intestines	_	_	_
	Liver	_	+ ^a^	+
	Skin	_	_	_
Pre-adults	Muscle	_	_	_
	Intestines	_	+	+
	Liver	_	+ ^a^	+
	Skin	_	_	_
Adults	Muscle	_	+	+
	Gonads	_	+	+
	Intestines	_	_	_
	Liver	_	+	+
	Skin	_	_	_
*Artemia* sp.	nauplii	_	_	_
Microalgae *mix*		_	_	_
Feed (1)		_	_	_
Feed (2)		_	_	_
TTX	STD	0.2	+ ^b^	+
Vehicule	0.1 N HCl	_	_	_

^a^ Rear limb paralysis and mild dyspnea; ^b^ Rear limb paralysis, mild dyspnea, convulsions, jumping, and death. * Viscera: Liver and intestines.

TTX transfer/accumulation profiles in the puffer fish body have been previously studied [27]. Kono *et al.* [28] studied non-toxic, cultivated juvenile *Fugu niphobles* fed a diet containing highly toxic natural *Fugu poecilonotus* liver for 30 days. They found that a part of the TTX accumulated in the liver from the diet was transferred to the skin. Wang *et al.* [29] studied toxin accumulation in hybrid specimens of puffer fish, *Takifugu niphobles* × *T.* *rubripes* and transfer/accumulation profiles in the puffer fish body. They find that part of the TTX is first accumulated in the liver and then transferred/accumulated in the skin in male specimens and in the ovary in female specimens. TTX that is intramuscularly administered to nontoxic, cultivated *T. rubripes* was rapidly transferred from the muscle via the blood to other organs. Little TTX was retained in the liver, and most (>96%) of the toxin remained in the skin. Immunohistochemical assay revealed that the toxin accumulated in the skin was localized in the basal cells of the epidermal layer [30].

The low toxicity of puffer fish cultivated under controlled conditions is safe for human consumption. The extracts of tissue from these nontoxic puffer fish could be used to enrich culture media for TTX-producing bacteria [31]. With increasing interest in applying TTX as an analgesic and anesthetic, further studies are recommended that focus on manipulating toxicity of puffer fish to determine potential medical uses.

In our study, the origin of the toxin was not determined and still remains an open question. Various researchers [9,11,32,33,34,35,36,37,38,39,40] consider some TTX-producing bacteria, such as *Vibrio*, *Pseudomonas*, *Schewanella*, *Microbacterium*, *Serratia*, *Nocardiopsis*, *Bacillus*, *Actinomycetes*, *Aeromonas*, and *Lysinibacillus* may be involved in puffer fish toxicity [33,34,35,36,39,40,41,42,43,44]. The biological role of TTX has been described as a defense mechanism [45] and also as a pheromone [46]. Saito *et al.* [47] reported evidence about attraction between the puffer fish *T. rubripes* fed food containing TTX.

Cultivation of innocuous puffer fish *T. rubripes* and *F. niphobles* have been reported [10,11,13,15,20]. However, other authors reported low quantities of TTX in these species in embryos and adult intestine [16,18,19]. The TTX content was analyzed by the monoclonal antibody method [18], which is more sensitive (nanograms) than the MBA used by Matsui *et al.* [13]. In our study, TTX was detected by MS-ESI, a highly sensitive method. Toxicity of cultivated puffer fish species is summarized in Table 2.

**Table 2:** Toxicity of cultured puffer fish.

Species	Toxicity	Analysis	Country	Reference
*Fugu niphobles*	<1 MU	MBA	Japan	[14]
	9–20 ng (<1 MU)	EIA	Japan	[18]
*Takifugu rubripes*	<1 MU	MBA	Japan	[15]
	0.1–0.2 MU	MBA, LC-FLD	Japan	[16]
	0.1–130 ng (<1 MU)	EIA	Japan	[17]
	2–10 MU			
	<10 MU–624 MU	MBA, LC-MS	Japan	[10,11,20,48]
		MBA	Taiwan	[19]
*Takifugu obscures T. rubripes*, *T. obscurus*, *T. floridus*, *T. bimaculatus*	<10 MU	MBA	China	[49]
	<1 MU	MBA	Korea	[50]
	<10 MU–125 MU	MBA	China	[26]
*Sphoeroides annulatus*	<1 MU	MBA, ESI-MS	Mexico	This study

MBA: Mouse bioassay; MU: Mouse unit (equivalent to 0.2 µg of TTX/g); HPLC-FLD: High performance liquid chromatography-fluorescence detector; EIA: Indirect competitive enzyme immunoassay; LC-MS: Liquid chromatography-mass spectrometry; ESI-MS: Electrospray interface-mass spectrometry.

## 3. Experimental Section

### 3.1. Puffer Fish Samples

**Figure 2:**
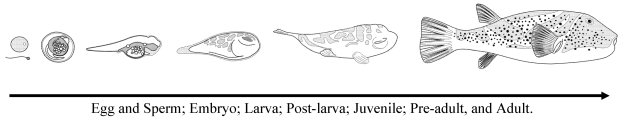
Analysis of toxicity of *Sphoeroides annulatus* at stages of development during cultivation.

Samples of sperm, eggs, embryos, larvae, post-larvae, juvenile, pre-adult, and adult specimens of *S. annulatus* were obtained from a closed hatchery culture system carried out at CIAD in Mazatlan Mexico (Figure 2, Table 3). Adult female fish were maintained under hatchery conditions and examined for egg maturity, based on oocyte size by gonadal biopsy. Fish with oocytes larger than 0.5 mm were hormone-treated with LHRHa over 48 h [24]. Three days after the hormone treatment, eggs and sperm were obtained by manual stripping; fecundation was induced by mixing eggs and sperm in seawater. Fertilized eggs were incubated in 600 L tanks with UV-treated and cartridge (20 μm)-filtered seawater at 28 °C. After 72 h, the eggs started to hatch and at day 4 post-hatch (DPH), the yolk sac of fish larvae was completely absorbed. First feeding of larvae started on DPH 4 with a mix of microalgae (*Nannochloropsis* sp., *Isochrysis* sp.) at a density of 100,000 cell/mL and rotifers (*Brachionus rotundiformis*) at a density of 5–10 rotifers/mL until DPH 16. *Artemia* nauplii was fed to the fish from DPH 16 through 29 at a density of 1–5 artemia/mL. Weaning on an artificial microdiet started at day 29 and ended at DPH 34. The artificial microdiet diet was fed in excess. It had a proximate composition of 49.5% protein, 8.8% lipid, 9.7% ash, and 92.4% dry matter. It was prepared with *Artemia* decapsulated cysts (80% total protein) and fish meal (20% total protein) as protein sources [51]. The microdiet was offered to the fish four times each day; the particle size was increased according to the fish size, ranging from 150–300 to 300–500 µm.

**Table 3:** Samples of *Sphoeroides annulatus* and feeds analyzed for TTX content.

Sample/development	Amount (weight or organisms)/age (days or months)	Characteristics (Size: mm; Weight: g)
Sperm	3 g	From 2 males born in captivity, 36 months
Eggs	20 g	From one female born in captivity, 36 months (246 mm and 620 g)
Embryo	10 g; 2 days after fecundation	Obtained in June 2004
Larva	5 g; 2 days after eclosion	Obtained in June 2004
Post-larva	150 organisms (1.5 months)	(average: 13.2 ± 2.14 mm and 1 ± 0.27 g)
Juvenile	70 organisms (4 months) ^1^	(average: 47.16 ± 2.85 mm and 3.29 ± 0.73 g)
Juvenile	6 organisms (16 months) ^1^	(average: 105.63 ± 8.96 mm and 34.04 ± 3.9 g)
Pre-adult	6 organisms (20 months) ^2^	(average: 172.5 ± 10.36 mm and 137.54 ± 37.37 g)
Pre-adult	5 organisms (24 months) ^2^	(average: 173.0 ± 13.50 mm and 149.6. ± 8.15 g)
Adults	5 organisms (36 months) ^3^	(average: 215.5 ± 12.15 mm and 260.2 ± 22.45 g) Obtained in 2007
*Artemia* sp. (nauplii)	10 g	Obtained in June 2004
Microalgae mix	10 g	Obtained in June 2007
Feed (1)	10 g	Produced in July 2004
Feed (2)	10 g	Produced in July 2007

^1^ Dissected: Viscera and muscle; ^2^ Intestines, liver, muscle, skin; ^3^ Gonad, intestines, liver, muscle, skin.

After the fish reached 1 g, fish were transferred to 3 m^ 3^ circular fiber glass tanks where feeding continued with a shrimp commercial feed with protein content of 32%. Seawater was maintained at normal seawater temperature and salinity of the season, which varied from 21.2 to 29.4 °C and from 32.6 to 35.1 ppt salinity. Samples of the different fish developmental stages, live food, and artificial feeds used were frozen at −20 °C and shipped on ice to the CIBNOR for toxin analysis.

### 3.2. Toxicity Assay

For the mouse bioassay, the fish and tissue samples were homogenized in 1:1 proportion with 0.1 N HCl with a blender, boiled for 5 min, and adjusted to pH 3 with 1 N HCl. The supernatant containing the toxin was obtained by centrifugation at 1100× *g* for 5 min and stored at 4 °C until used. CD-1 male mice weighing 18–23 g each, in groups of three, were injected intraperitoneally with aliquots of the toxin extract. Toxicity was determined by the average surviving time, according to a standard dose-lethal time plot prepared with commercial TTX (Sigma, St Louis, MO, USA). In this assay, one mouse unit of TTX, defined as the amount of toxin necessary to kill the mouse in 7–15 min, corresponded to 0.2 µg TTX. Toxicity is expressed as the concentration of TTX-equivalents (µg/g fish sample) [5].

### 3.3. ESI-MS Analysis

The electrospray ionization-mass spectrometry (ESI-MS) analysis (1100 spectrometer, Agilent Technologies, Santa Clara, CA, USA) was used to determine TTX, following the recommendations of Shoji *et al.* [52]. For this, the samples were centrifuged at 10,000× *g* for 15 min (Beckman-Coulter microfuge E, Brea, CA, USA). The supernatant was filtered (0.22 µm millipore membrane) and mixed with ammonium formate in 4:1 proportion (40 µL extract and 10 µL ammonium formate) before analysis in the ESI-MS system. 

Compound mass spectrum list report-MS. Acquisition parameter: mass range mode (Std/Normal); ion polarity (positive): ion source type (ESI); dry temperature (set) (350 °C); nebulizer (set) (40.00 psi); dry gas (set) (9.00 L/min); trap drive (33.2); skim 1 (33.8 V); skim 2 (6.0 V); octopole RF amplitude (150.0 V pp); capillary exit (106.6 V); capillary exit offset (72.8 V); scan begin (300 *m/z*); scan end (400 *m/z*); averages (8 spectra); maximum acceleration time (200,000 µs); ICC target (30,000).

## 4. Conclusions

In summary, cultivated bullseye puffer fish appear to be innocuous, if the lethal dose of TTX is 0.5–2.0 mg for a 50 kg person [9]. We found that the amount of TTX in cultivated *S. annulatus* tissues is 1000 times lower.
